# Trends in pneumothorax mortality in England (2004–2023): a population-based observational study

**DOI:** 10.1016/j.lanepe.2026.101632

**Published:** 2026-03-04

**Authors:** Xiaomin Zhong, Eva J.A. Morris, Raph Goldacre, Rob J. Hallifax

**Affiliations:** a Applied Health Research Unit, Big Data Institute, Li Ka Shing Centre for Health Information and Discovery, Nuffield Department of Population Health, University of Oxford, Oxford, UK; b Oxford Centre for Respiratory Medicine, Oxford University Hospitals NHS Foundation Trust, Oxford, UK

**Keywords:** Pneumothorax, Mortality, Epidemiology, COVID

## Abstract

**Background:**

Monitoring pneumothorax mortality trends helps quantify disease burden, identify high-risk groups, assess guideline impacts, and inform resource planning. This study examined spontaneous pneumothorax (SP) mortality trends in England.

**Methods:**

We conducted a population-based observational study using linked national death registrations from the Office for National Statistics and Hospital Episode Statistics Admitted Patient Care (HES-APC) from 1st January 2004 to 31st December 2023. SP-related mortality was any mention of spontaneous pneumothorax on the death certificate; SP-specific was spontaneous pneumothorax as the underlying cause. In this study, primary and secondary spontaneous pneumothorax follow a modified definition from conventional guidelines and included patients (of any age) with and without documented chronic lung disease respectively. We classified in-hospital deaths as primary or secondary spontaneous pneumothorax using chronic lung disease recorded on the death certificate or in linked HES-APC records.

**Findings:**

We identified 6,442 spontaneous pneumothorax-related deaths, including 5,055 in-hospital deaths. Among in-hospital deaths, 4,093 (81.0%) had chronic lung disease and 962 (19.0%) had no recorded lung disease. From 2004 to 2019, the mean annual spontaneous pneumothorax-related mortality rate was 6.4 per 1,000,000 and declined year on year (IRR 0.986, 95% CI 0.980–0.991). Rates increased after 2019 and peaked at 8.1 per 1,000,000 in 2021, then fell to 6.9 per 1,000,000 in 2023. The excess in 2021 was largely COVID-related (2.3 per 1,000,000). In 2021, non-COVID mortality was similar to pre-2020 levels. From 2017 to 2023, spontaneous pneumothorax was certified as the underlying cause in 7.9% (215/2,735) of spontaneous pneumothorax-related death certificates, whereas it appeared in Part I (the section documenting the causal sequence of conditions leading directly to death) in 64% (1,750/2,735).

**Interpretation:**

In 2020, the COVID-19 pandemic interrupted a long-term reduction in spontaneous pneumothorax mortality. Relying solely on underlying cause of death substantially underestimates pneumothorax's overall contribution to mortality in England. Patients and clinicians should be aware that pneumothorax is not an entirely benign disease and remains a significant cause of mortality in individuals with and without underlying lung disease, particularly in those over 65 years of age.

**Funding:**

This work was supported by the NIHR Oxford Biomedical Research Centre and by Health Data Research UK.

**Research in context**

**Evidence before this study**

We searched PubMed from inception to 30th May 2025 for research on spontaneous pneumothorax (SP) mortality, trends, and outcomes. Our search terms included “spontaneous pneumothorax,” “primary pneumothorax,” “secondary pneumothorax,” “mortality,” and “epidemiology.” We found that many published studies relied on hospital readmission datasets or were single-centre investigations, and most were outdated. Only one national-level study from England, published over 25 years ago, reported an annual SP mortality of about 1.3 deaths per million men and 0.6 per million women. That work showed a marked increase in mortality among older adults (≥55 years) between 1960 and 1990, suggesting a higher risk for secondary spontaneous pneumothorax. However, it did not clearly differentiate between primary and secondary pneumothorax and preceded the COVID-19 era.

**Added value of this study**

By analysing two decades (2004–2023) of linked death registrations and hospital admissions data in England, this study provides an up-to-date perspective on spontaneous pneumothorax mortality. It is the first to distinguish primary and secondary spontaneous pneumothorax mortality using specific clinical coding, capturing both spontaneous pneumothorax-related (any mention of pneumothorax) and spontaneous pneumothorax-specific (underlying cause) deaths. Furthermore, it assesses how mortality patterns shifted before and after the COVID-19 pandemic. By quantifying and highlighting other key underlying causes in the chain of events leading to death (particularly chronic obstructive pulmonary disease (COPD) and COVID-19 infection), our findings address an important gap in the understanding of how these conditions influence spontaneous pneumothorax mortality.

**Implications of all the available evidence**

When considered alongside earlier research, our results indicate that older individuals and those with chronic lung disease face a substantially higher risk of death from spontaneous pneumothorax. These data reinforce the need for targeted prevention, timely intervention, and careful management in high-risk groups. The pandemic-related rise in spontaneous pneumothorax mortality also highlights the importance of incorporating pneumothorax complications into post-COVID respiratory care planning. By leveraging the broader evidence base, clinicians and policymakers can allocate resources more effectively, prioritise prompt treatment, and ultimately reduce SP-related mortality in the wake of COVID-19.

## Introduction

Spontaneous pneumothorax is a common pathology. Primary spontaneous pneumothorax (PSP) conventionally refers to patients with no underlying lung disease, whilst those with established lung pathology are classified as secondary spontaneous pneumothorax (SSP). There is a bimodal age-distribution with a first peak aged 15–34 years and an increasing incidence beyond 60 years in both males and females.^1^ Overall incidence of spontaneous pneumothorax is 17–24 per 100,000 population per annum for men and 1–6 per 100,000 for women.^1^, ^2^, ^3^, ^4^, ^5^ In England over the 50 years up to 2016, there was a small gradual increase in the incidence of inpatient admissions for spontaneous pneumothorax,^1^ but this has appeared to plateau after a peak of COVID-related pneumothoraces in 2020–21.^5^

In most cases, spontaneous pneumothorax takes a benign course: patients usually present with pain and/or breathlessness and treatment options range from conservative management to needle aspiration of air, insertion of an ambulatory Heimlich valve device, or chest drain insertion and admission to hospital. However, spontaneous pneumothorax is a potentially life-threatening condition, particularly in patients with underlying lung diseases such as chronic obstructive pulmonary disease (COPD) or interstitial lung disease (ILD).^6^

There is a lack of high-quality up-to-date population-based epidemiological studies on the mortality risk of patients admitted with spontaneous pneumothorax. Previous studies are from readmission datasets, single-centre retrospective studies or are, simply, out of date. The only previous publication using English national data was published 25 years ago and did not robustly distinguish between PSP and SSP (rather just by age-criteria).^3^

This study aimed to investigate trends in the mortality of pneumothorax in England, by age, sex and comorbidity.

## Methods

### Ethical approval

Under the assessment of the NHS Health Research Authority, the use of linked Hospital Episode Statistics Admitted Patient Care (HES-APC) and mortality data in this study, conducted within the programme of epidemiological and health services research at the University of Oxford, does not need research ethics committee approval as it is anonymised data.

### Data sources and study design

We conducted a population-based time-series analysis of national civil death registrations in England from 1st January 2004 to 31st December 2023. To enhance the clinical information available at death, we linked these records to English national HES-APC data, which include all NHS inpatient and day-case episodes nationwide.

### Case identification and exclusions

English death certificates comprise two sections: Part I lists the causal sequence of events leading to death, while Part II records other contributing conditions. The underlying cause is selected from these entries based on ICD coding rules and clinical judgement. Although we could identify the underlying cause throughout the entire study period, only records from 2017 onward allowed us to distinguish whether other mentions appeared in Part I or Part II. We included all death certificates that mentioned spontaneous pneumothorax (International Statistical Classification of Diseases, 10th Revision (ICD-10) J93) in any position (underlying cause or anywhere else in Part I or Part II). Based on the death record and the linked HES-APC record, we excluded any cases that also listed iatrogenic pneumothorax (J95.8) or traumatic pneumothorax (S22, S27, S42), as well as cases in which the individual was aged under 15 years at death. This approach followed the same strategy as in our previous studies.^1^^,^^5^

### Outcome definitions

The primary outcome was spontaneous pneumothorax-related death. These included cases in which spontaneous pneumothorax was listed anywhere on the death certificate, not just the underlying cause. Because causes of out-of-hospital deaths are less accurately ascertained, particularly those without any previous hospital admission record, we also evaluated in-hospital spontaneous pneumothorax-related deaths separately and further classified them as primary spontaneous pneumothorax (PSP) or secondary spontaneous pneumothorax (SSP). In this study, SSP and PSP follow a modified definition from conventional guidelines and included patients (of any age) with and without documented chronic lung disease respectively. SSP was assigned if the death certificate or linked HES data recorded any chronic lung disease, as defined by the 73 ICD-10 codes listed in eTable S1 of the Supplementary Material. All other cases were classified as PSP. This is the same methodology of classification of PSP and SSP as utilised in our previous studies.^1^^,^^5^ The coding of chronic respiratory comorbidities in HES is among the most robustly documented: the accuracy for chronic lung disease is 86%).^7^ Therefore, misclassification of PSP and SSP is likely to be low. Mortality records listing COVID-19 (U07.1) alongside spontaneous pneumothorax were labelled as COVID-related and analysed separately.

### Statistical analysis

Annual mortality rates were calculated per 1,000,000 population, using Office for National Statistics mid-year estimates as denominators^8^ and the 2013 European Standard Population for age-standardisation.^9^ Rates were produced overall and separately for in-hospital spontaneous pneumothorax overall, PSP and SSP, with and without COVID-related deaths. Age-specific rates were reported by sex and by broader age groups (15–34, 35–49, 50–64, ≥65 years); age-standardisation was conducted within the broad age groups.

To assess temporal trends, we fitted over-dispersed Poisson regression models adjusted for age (5-year groups) and sex. Based on interrupted time series methods described by Bernal et al., year was entered as a continuous variable, while pandemic and post-pandemic years were modelled categorically (pre-2020, 2020, 2021, 2022, 2023; pre-2020 was the reference), providing incidence rate ratios (IRRs) with 95% confidence intervals.^10^ To examine changes in the distribution of underlying causes over time, we listed the leading underlying causes of in-hospital spontaneous pneumothorax-related deaths in 2004–2007 and compared them with those in 2016–2019, and 2020–2023, repeating the comparison separately for in-hospital PSP and SSP. To verify consistency between hospital records and death certificates, we also listed the leading primary diagnosis of the hospitalisation preceding death among in-hospital spontaneous pneumothorax cases, and repeated this for in-hospital PSP and SSP. Given the rarity of in-hospital spontaneous pneumothorax-related deaths, particularly PSP, and the predominance of deaths among older individuals with multimorbidity, we repeated the analyses of leading underlying causes and primary diagnoses in the subgroup aged 50 years or over.

In a secondary analysis, to prospectively estimate the risk of in-hospital death following SP, we assembled a cohort of emergency admissions with eligible spontaneous pneumothorax and used these admissions as the denominator. We reported the in-hospital risk of (i) death from any cause (where spontaneous pneumothorax might or might not have been recorded as a cause of death), (ii) spontaneous pneumothorax-related death (pneumothorax mentioned anywhere on the death certificate), and (iii) spontaneous pneumothorax-specific death (pneumothorax as the underlying cause). Admissions were classified as PSP or SSP, and we additionally distinguished whether pneumothorax was recorded as the primary diagnosis (i.e. the main reason of the emergency admission or as a secondary diagnosis. Each analysis was stratified by co-existing COVID-19. For those with PSP and a diagnosis of COVID-19, the preferred description was “COVID-19 without known lung disease SP,” so we will use this throughout where applicable. Definitions and exclusions mirrored those used elsewhere (excluding iatrogenic or traumatic pneumothorax and persons aged <15 years). Risks were summarised overall and by age group and sex.

To evaluate complications potentially related to chest tube insertion, we calculated the proportions with procedure codes indicating invasive mechanical ventilation or non-invasive ventilation (OPCS-4 E85; E85.1 for NIV) among in-hospital SP, PSP and SSP, and repeated these analyses separately for COVID-related and non-COVID deaths. Analyses were conducted in R 4.3.0.

### Role of the funding source

The funders of this study had no role in study design, data collection, data analysis, data interpretation, writing the report or decision to submit.

## Results

Between January 2004 and December 2023, we identified 7,187 deaths in which an ICD-10 code for spontaneous pneumothorax was recorded on the death certificate. Of these, 6442 met the study definition of a spontaneous pneumothorax-related death. Of the spontaneous pneumothorax-related deaths, 5,055 occurred in hospital. Among in-hospital spontaneous pneumothorax-related deaths, 962 (962/5,055, 19.0%) were classified as primary spontaneous pneumothorax and 4,093 (4,093/5,055, 81.0%) as secondary spontaneous pneumothorax. Men accounted for 3,395 deaths (3,395/5,055, 67.2%), and the mean age at death was 74.1 years (SD 12.7). Case selection is shown in eFigure S1.

**Fig. 1:**
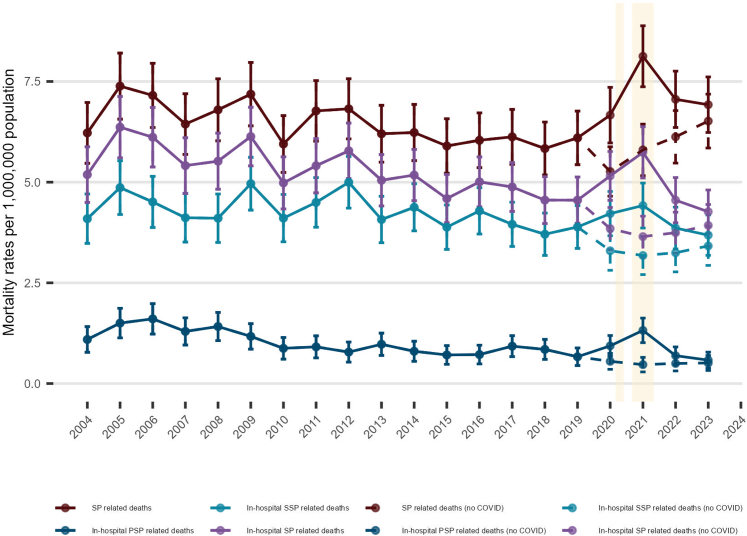
**Age-standardised Mortality Rates for Spontaneous Pneumothorax (SP-specific and in-hospital) in England (Jan 2004–Dec 2023).** Footnote: Points show directly age-standardised mortality rates per 1,000,000 population (standardised to the 2013 European Standard Population). Population denominators are Office for National Statistics (ONS) mid-year estimates of the usual resident population of England. Error bars denote 95% confidence intervals based on a Poisson approximation to the variance of the directly standardised rate; intervals are truncated at zero where necessary. Shaded bands indicate the first and second waves of the COVID-19 pandemic in England. Dashed lines (“no COVID”) exclude deaths involving COVID-19 as defined in the Methods. ∗PSP denotes SP with no recorded chronic lung disease in ONS/Hospital Episode Statistics.

Fig. 1 shows annual age-standardised mortality rates per million population for spontaneous pneumothorax. Between 2004 and 2019, the mean annual rate was 6.4 per million overall. For in-hospital SP-related deaths, the mean annual rates were 5.3 (SD 0.6, all spontaneous pneumothorax), 1.0 (SD 0.3, primary spontaneous pneumothorax) and 4.3 (SD 0.4, secondary spontaneous pneumothorax). Over this period the rate declined (annual incidence-rate ratio, IRR = 0.986, 95% CI 0.980–0.991). After 2019 the trend reversed: the overall spontaneous pneumothorax rate rose from 6.1 per million in 2019 to 6.7 in 2020, peaking at 8.1 in 2021. Relative to the pre-2020 period, the 2021 IRR was 1.31 (95% CI 1.17–1.46) for overall spontaneous pneumothorax; for in-hospital deaths the IRR was 1.17 (95% CI 1.03–1.32) for SP (all), 1.51 (95% CI 1.17–1.94) for primary spontaneous pneumothorax, and 1.06 (95% CI 0.91–1.23) for secondary spontaneous pneumothorax (see eFigure S2). In 2023 the overall spontaneous pneumothorax rate fell to 6.9 per million. The pandemic-era increase was driven by COVID-related deaths: excluding COVID-19, the 2021 rates were 5.8 per million for overall spontaneous pneumothorax, and for in-hospital spontaneous pneumothorax were 3.7 (all), 0.5 (PSP) and 3.2 (SSP) per million (see eFigure S2).

**Fig. 2:**
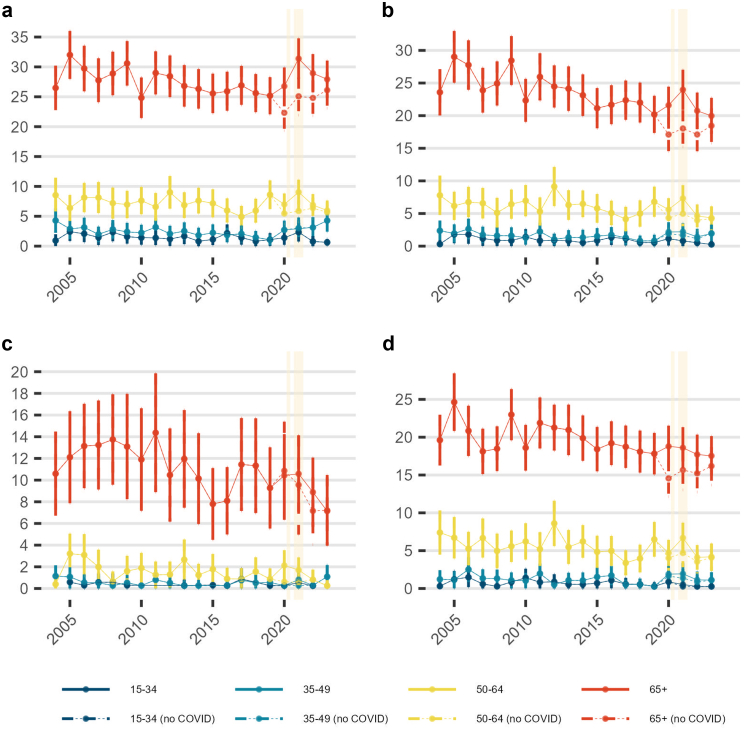
**Age-standardised mortality rates for spontaneous pneumothorax by age group (15–34, 35–49, 50–64, 65+) in England (Jan 2004–Dec 2023). Panels: (a) All spontaneous pneumothorax deaths, (b) In-hospital spontaneous pneumothorax, (c) In-hospital PSP, (d) In-hospital SSP.** Footnote: Points show directly age-standardised mortality rates per 1,000,000 population (standardised to the 2013 European Standard Population). Population denominators are Office for National Statistics (ONS) mid-year estimates of the usual resident population of England. Error bars denote 95% confidence intervals based on a Poisson approximation to the variance of the directly standardised rate and are truncated at zero where necessary. Dashed lines (“no COVID”) exclude deaths involving COVID-19 as defined in the Methods. Shaded bands indicate the first and second COVID-19 waves in England. The overall (all-ages) series is omitted; only the four broader age groups are shown. ∗PSP denotes SP with no recorded chronic lung disease in ONS/Hospital Episode Statistics.

**Fig. 3:**
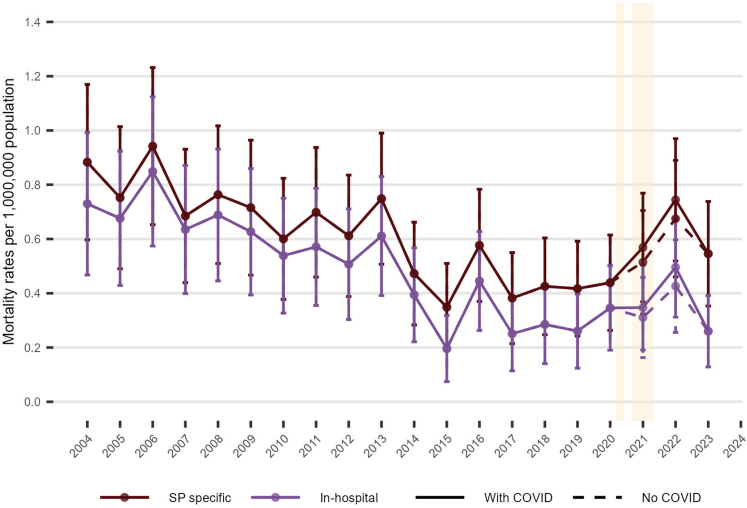
**Age-standardised mortality rates for spontaneous pneumothorax (SP-specific and in-hospital SP-specific) in England, Jan 2004–Dec 2023.** Footnote: Points show directly age-standardised mortality rates per 1,000,000 population (standardised to the 2013 European Standard Population). Population denominators are Office for National Statistics (ONS) mid-year estimates of the usual resident population of England. Error bars denote 95% confidence intervals based on a Poisson approximation to the variance of the directly standardised rate; intervals are truncated at zero where necessary. Colours distinguish SP-specific vs in-hospital SP-specific deaths; line type indicates inclusion of COVID-19 (solid) or exclusion (“no COVID”, dashed). Shaded bands indicate national lockdown periods in England.

Fig. 2 shows age-standardised mortality by age group for spontaneous pneumothorax, in-hospital spontaneous pneumothorax, in-hospital primary spontaneous pneumothorax, and in-hospital secondary spontaneous pneumothorax. Mortality rose with age, with the highest rates in those aged ≥65 years, intermediate in 50–64, and lowest in 35–49 and 15–34; the pandemic-era increase was concentrated in the ≥65-year group, while the no-COVID series changed only modestly. Across the study period, mean annual rates per 1,000,000 were as follows: overall spontaneous pneumothorax, 27.8 (SD 2.1, ≥65), 7.2 (SD 1.1, 50–64), 2.6 (SD 0.8, 35–49), 1.5 (SD 0.6, 15–34); in-hospital spontaneous pneumothorax, 23.6 (SD 2.6), 6.1 (SD 1.2), 1.7 (SD 0.5), 0.9 (SD 0.4) (same order); in-hospital primary spontaneous pneumothorax, 11.0 (SD 2.0), 1.5 (SD 0.8), 0.5 (SD 0.3), 0.3 (SD 0.3); and in-hospital secondary spontaneous pneumothorax, 19.6 (SD 1.9), 5.6 (SD 1.3), 1.2 (SD 0.6), 0.7 (SD 0.4). As shown in eFigure S3, men had higher mortality than women across every age group and definition. Fig. 3 shows a long downward trend in spontaneous pneumothorax-specific and in-hospital spontaneous pneumothorax-related mortality through the 2000s and early 2010s, a rise in 2020–2022, and partial reversion by 2023. For spontaneous pneumothorax-specific mortality, the mean annual rate between 2004 and 2019 was 0.6 (SD 0.2) deaths per million. The rate fell from 0.9 per million in 2004 to 0.4 per million in 2015–2019 (SD 0.1, minimum 0.3 in 2015), before increasing during the pandemic. Additionally, eTable S2 summarises where pneumothorax was recorded on death certificates issued between 2017 and 2023 (the earliest year for which position data were available). Overall, spontaneous pneumothorax was certified as the underlying cause in 7.9% (215/2,735) of spontaneous pneumothorax-related death certificates, whereas it appeared in Part I (the section documenting the causal sequence of conditions leading directly to death) in 64% (1,750/2,735).

In our secondary analysis, we identified 211,722 emergency admissions with eligible spontaneous pneumothorax (Table 1). Overall, 12.9% (27,346/211,722) of admissions ended in in-hospital death; however, only 2.1% (4,516/211,722) of these deaths were certified as spontaneous pneumothorax-related and just 0.2% (417/211,722) were spontaneous pneumothorax-specific. Restricting to non-COVID admissions where spontaneous pneumothorax was the primary diagnosis, all-cause in-hospital mortality was 1.7% (1,227/72,230) for PSP and 6.9% (3,467/50,431) for SSP; spontaneous pneumothorax-specific deaths were 0.1% in both. SSP consistently carried higher risk than PSP; for example, pneumothorax-related mortality after admission for spontaneous pneumothorax as the primary diagnosis was 0.5% (356/72,230) in non-COVID PSP vs 3.4% (1,737/50,431) in non-COVID SSP. Mortality was higher when spontaneous pneumothorax was recorded as a secondary rather than a primary diagnosis: among men with non-COVID SSP, the adjusted all-cause risk was 26.7% (95% CI 26.1–27.2) with spontaneous pneumothorax secondary vs 7.0% (95% CI 6.8–7.3) when primary, with SP-related risk 3.9% (95% CI 3.7–4.2) vs 3.6% (95% CI 3.4–3.8). Risks rose steeply with age, and age-adjusted risks in men were higher than in women (Table 1).

**Table 1:** In-hospital mortality among emergency admissions for spontaneous pneumothorax, by COVID status and primary vs secondary diagnosis.

Type	Admissions	All-cause deaths, n (%)	SP-related deaths, n (%)	SP-specific deaths, n (%)
non-COVID PSP (Primary position)	72,230	1,227 (1.7%)	356 (0.5%)	103 (0.1%)
non-COVID PSP (Secondary position)	41,991	9,362 (22.3%)	613 (1.5%)	161 (0.4%)
non-COVID SSP (Primary position)	50,431	3,467 (6.9%)	1,737 (3.4%)	70 (0.1%)
non-COVID SSP (Secondary position)	41,272	10,804 (26.2%)	1,567 (3.8%)	78 (0.2%)
COVID without known lung disease SP	3,276	1,510 (46.1%)	93 (2.8%)	<5 (0.1%)
COVID-related SSP	2,522	976 (38.7%)	150 (5.9%)	<5 (0.0%)
All SP (total)	211,722	27,346 (12.9%)	4,516 (2.1%)	417 (0.2%)

In-hospital mortality after emergency admission with spontaneous pneumothorax (non-COVID), adjusted risks (%) and adjusted risk ratios by sex and age, stratified by pneumothorax type (PSP vs SSP) and diagnosis position (primary vs secondary)											
Group	Level	Admission	All cause			SP-related			SP-specific		
			Crude risk	Adjusted risk	Adjusted risk ratio	Crude risk	Adjusted risk	Adjusted risk ratio	Crude risk	Adjusted risk	Adjusted risk ratio
**Non-COVID Spontaneous pneumothorax without known lung disease, PSP**^a^**(Primary position)**											
Sex											
	Male	54,195	1.48%	1.59% (1.48%–1.70%)	0.82 (0.73–0.91)	0.46%	0.49% (0.43%–0.55%)	1.00 (0.80–1.25)	0.13%	0.14% (0.11%–0.17%)	0.97 (0.64–1.47)
	Female	18,035	2.36%	1.95% (1.77%–2.13%)	1.00	0.60%	0.49% (0.40%–0.58%)	1.00	0.18%	0.15% (0.10%–0.20%)	1.00
Age											
	15–34	38,177	0.07%	0.07% (0.04%–0.10%)	0.20 (0.12–0.32)	0.01%	0.01% (0.00%–0.02%)	0.10 (0.03–0.31)	0.00%	0.00% (0.00%–0.01%)	0.36 (0.02–5.75)
	35–49	13,721	0.35%	0.34% (0.25%–0.44%)	1.00	0.10%	0.10% (0.05%–0.16%)	1.00	0.01%	0.01% (0.00%–0.02%)	1.00
	50–64	8,083	2.02%	1.99% (1.68%–2.29%)	5.78 (4.19–7.96)	0.51%	0.50% (0.35%–0.66%)	4.93 (2.69–9.04)	0.05%	0.05% (0.00%–0.10%)	6.75 (0.75–60.36)
	65+	12,249	8.08%	8.02% (7.54%–8.50%)	23.32 (17.47–31.12)	2.42%	2.43% (2.16%–2.71%)	23.75 (13.90–40.57)	0.79%	0.79% (0.63%–0.95%)	108.75 (15.17–779.78)
**Non-COVID Spontaneous pneumothorax without known lung disease, PSP (Secondary position)**											
Sex											
	Male	25,353	22.07%	22.40% (21.89%–22.91%)	1.01 (0.98–1.05)	1.48%	1.52% (1.37%–1.67%)	1.10 (0.94–1.29)	0.37%	0.39% (0.31%–0.47%)	1.03 (0.75–1.41)
	Female	16,638	22.63%	22.14% (21.52%–22.76%)	1.00	1.43%	1.38% (1.20%–1.55%)	1.00	0.40%	0.38% (0.29%–0.47%)	1.00
Age											
	15–34	4,628	10.85%	10.68% (9.79%–11.56%)	0.63 (0.57–0.70)	0.45%	0.44% (0.25%–0.63%)	0.59 (0.35–1.01)	0.09%	0.08% (0.00%–0.16%)	0.81 (0.22–3.00)
	35–49	4,773	16.99%	16.87% (15.82%–17.93%)	1.00	0.75%	0.74% (0.50%–0.99%)	1.00	0.10%	0.10% (0.01%–0.19%)	1.00
	50–64	8,360	19.78%	19.90% (19.05%–20.76%)	1.18 (1.09–1.27)	0.90%	0.91% (0.70%–1.11%)	1.22 (0.82–1.81)	0.20%	0.21% (0.11%–0.31%)	2.02 (0.75–5.47)
	65+	24,230	26.39%	26.44% (25.89%–26.99%)	1.57 (1.47–1.67)	1.99%	2.00% (1.82%–2.17%)	2.68 (1.91–3.76)	0.56%	0.56% (0.47%–0.66%)	5.49 (2.25–13.38)
**Non-COVID Spontaneous pneumothorax with known lung disease, SSP (Primary position)**											
Sex											
	Male	35,193	7.02%	7.03% (6.77%–7.29%)	1.08 (1.01–1.16)	3.57%	3.58% (3.39%–3.78%)	1.15 (1.03–1.27)	0.14%	0.14% (0.10%–0.18%)	1.13 (0.67–1.92)
	Female	15,238	6.55%	6.52% (6.14%–6.91%)	1.00	3.15%	3.13% (2.86%–3.40%)	1.00	0.12%	0.13% (0.07%–0.18%)	1.00
Age											
	15–34	7,581	0.45%	0.44% (0.29%–0.59%)	0.29 (0.20–0.44)	0.16%	0.16% (0.07%–0.24%)	0.39 (0.19–0.78)	0.03%	0.03% (0.00%–0.06%)	1.39 (0.13–15.32)
	35–49	5,570	1.47%	1.49% (1.17%–1.81%)	1.00	0.39%	0.40% (0.23%–0.57%)	1.00	0.02%	0.02% (0.00%–0.05%)	1.00
	50–64	11,013	4.74%	4.72% (4.32%–5.11%)	3.17 (2.52–4.00)	2.18%	2.18% (1.90%–2.45%)	5.45 (3.52–8.42)	0.05%	0.04% (0.01%–0.08%)	2.46 (0.29–21.06)
	65+	26,267	10.77%	10.84% (10.46%–11.21%)	7.29 (5.86–9.06)	5.57%	5.59% (5.31%–5.87%)	14.00 (9.20–21.31)	0.24%	0.24% (0.18%–0.30%)	13.06 (1.81–94.23)
**Non-COVID Spontaneous pneumothorax with known lung disease, SSP (Secondary position)**											
Sex											
	Male	25,121	26.79%	26.69% (26.14%–27.23%)	1.05 (1.02–1.09)	3.98%	3.94% (3.71%–4.18%)	1.11 (1.00–1.22)	0.20%	0.19% (0.14%–0.25%)	1.06 (0.67–1.68)
	Female	16,151	25.22%	25.38% (24.72%–26.05%)	1.00	3.52%	3.56% (3.28%–3.85%)	1.00	0.18%	0.18% (0.12%–0.25%)	1.00
Age											
	15–34	2,376	10.52%	10.34% (9.13%–11.56%)	0.58 (0.51–0.67)	0.93%	0.90% (0.52%–1.27%)	0.61 (0.37–1.01)	0.08%	0.08% (0.00%–0.19%)	0.85 (0.14–5.06)
	35–49	3,096	17.76%	17.72% (16.38%–19.06%)	1.00	1.49%	1.48% (1.06%–1.90%)	1.00	0.10%	0.10% (0.00%–0.21%)	1.00
	50–64	8,786	22.64%	22.58% (21.71%–23.46%)	1.27 (1.17–1.39)	2.55%	2.54% (2.21%–2.86%)	1.71 (1.25–2.35)	0.07%	0.07% (0.01%–0.12%)	0.70 (0.18–2.82)
	65+	27,014	29.67%	29.73% (29.19%–30.28%)	1.68 (1.55–1.81)	4.72%	4.74% (4.49%–5.00%)	3.21 (2.40–4.29)	0.25%	0.25% (0.19%–0.31%)	2.59 (0.82–8.24)

Admissions: number of eligible emergency admissions in which spontaneous pneumothorax (the 10th revision of the International Classification of Diseases (ICD-10) J93) was recorded in any diagnosis during the stay. Primary: spontaneous pneumothorax (ICD-10 J93) recorded as the main diagnosis for the admission. Secondary: spontaneous pneumothorax (ICD-10 J93) recorded only as an additional/secondary diagnosis (i.e., not the main diagnosis). COVID-related: a COVID-19 diagnosis (ICD-10 U07.x) recorded as a coexisting condition during the admission. Non-COVID: no COVID-19 diagnosis recorded during the admission. All-cause deaths: deaths occurring in hospital from any cause. SP-related deaths: in-hospital deaths where spontaneous pneumothorax was listed anywhere on the death certificate. SP-specific deaths: in-hospital deaths where spontaneous pneumothorax was certified as the underlying cause of death.

SP, spontaneous pneumothorax; PSP, primary spontaneous pneumothorax; SSP, secondary spontaneous pneumothorax. “Primary position” indicates SP coded as the primary diagnosis of the admission; “Secondary position” indicates SP coded as a non-primary diagnosis during the admission. All-cause death = any in-hospital death; SP-related death = death certificate mentions SP anywhere; SP-specific death = SP as the underlying cause of death. Adjusted risks are model-based predicted probabilities (percent) from regression adjusted for sex and age group within each stratum; adjusted risk ratios are relative to the reference categories shown (female for sex; ages 35–49 for age). Values are shown with 95% confidence intervals in parentheses. Non-COVID excludes admissions or deaths with COVID-19 (U07.1). In Hospital Episode Statistics (HES) or Office for National Statistics (ONS), sex is a data field that uses a code to define the biological sex of the patient. Ethnicity information was not available for this study and is therefore not reported.

a PSP denotes SP with no recorded chronic lung disease in ONS/HES.

**Fig. 4:**
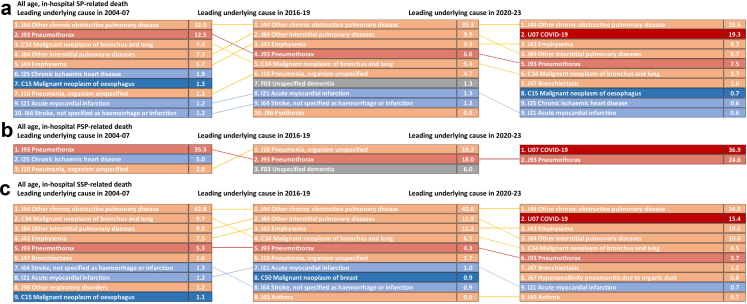
**Leading underlying causes of in-hospital spontaneous pneumothorax-related deaths in England, 2004–07, 2016–19 and 2020–23 (all ages). Panels: (a) All spontaneous pneumothorax, (b) PSP, (c) SSP.** Footnote. Bars list the top underlying causes by the 10th revision of the International Classification of Diseases (ICD-10) three-character code; values are the percentage of in-hospital SP-related deaths within each period. Colours denote cause groups:  respiratory diseases (including lung cancer);  circulatory diseases;  other malignant neoplasms (excluding lung);  COVID 19;  Pneumothorax; and  Other diseases. Dotted lines trace changes in rank between periods. Causes with fewer than 5 deaths in a period are not shown.

Fig. 4 compares the underlying causes of in-hospital spontaneous pneumothorax-related deaths across 2004–07, 2016–19, and 2020–23. Overall, chronic obstructive pulmonary disease (COPD; J44) remained the most common underlying cause (32.5% in 2004–07, 35.3% in 2016–19, 28.6% in 2020–23). Pneumothorax itself (J93) was less often recorded as the underlying cause in 2020–23 than earlier, while COVID-19 (U07) emerged prominently in 2020–23 (19.3%). Within PSP, the leading underlying cause shifted from pneumothorax (J93) in 2004–07 (35.3%) to pneumonia (J18) in 2016–19 (19.2%), and then to COVID-19 (U07) in 2020–23 (36.9%), with pneumothorax second (24.6%). Within SSP, COPD (J44) remained dominant throughout (42.8% in 2004–07, 42.6% in 2016–19, 34.8% in 2020–23), with important contributions in 2020–23 from emphysema (J43, 10.6%), other interstitial pulmonary diseases (J84, 10.6%), and COVID-19 (U07, 15.4%). Cardiovascular disease was the leading non-respiratory category across periods, with chronic ischaemic heart disease (I25) and acute myocardial infarction (I21) appearing consistently among the top underlying causes. Consistent with these patterns, eTable S3 shows similar rankings for the hospital primary diagnosis. Among all in-hospital SP-related deaths, pneumothorax (J93) was the leading primary diagnosis (i.e. the main reason for admission) in each period (34.3% in 2004–07, 42.3% in 2016–19, 36.6% in 2020–23), with pneumonia (J18) also prominent (9.9%, 16.7%, 10.9%) and COVID-19 (U07) appearing in 2020–23 (14.3%). In in-hospital PSP, the leading primary diagnosis moved from pneumothorax (J93, 24.4%) in 2004–07 to pneumonia (J18, 22.2%) in 2016–19; In 2020–23 the leading type was “COVID without known lung disease spontaneous pneumothorax” (U07, 32.8%). In in-hospital SSP, pneumothorax (J93) remained the leading primary diagnosis across all periods (37.5%, 46.9%, 41.6%). When limiting analyses to age 50 years and older, 896 of 962 (93.1%) in-hospital primary spontaneous pneumothorax deaths and 3,937 of 4,093 (96.2%) in-hospital SSP deaths remained, and the underlying-cause and primary-diagnosis patterns were almost unchanged (eTables S4 and S5).

eTable S6 shows that, among deaths in hospital for patients without known underlying lung disease, individuals with a concurrent diagnosis of COVID-19 had much higher use of breathing support than those without (46/82, 56.1% vs 120/880, 13.6%; approximately four-fold higher) and a higher NIV rate (10/82, 12.2% vs 62/880, 7.0%). Among those with known underlying lung disease who died in hospital, breathing support was also more frequent in those with COVID-19 (64/166, 38.6% vs 726/3,927, 18.5%; approximately two-fold higher), whereas NIV was slightly lower (17/166, 10.2% vs 559/3,927, 14.2%).

## Discussion

To the best of our knowledge, we present the largest study of pneumothorax mortality in England to date. Our study highlights a significant long-term decline in mortality rates from spontaneous pneumothorax between 2004 and 2019, followed by a marked increase after the onset of the COVID-19 pandemic in 2020. The pre-pandemic downward trend in spontaneous pneumothorax-related mortality is consistent with improvements in respiratory care and service delivery over time, alongside changes in population structure and the impact of COVID-19. However, the sharp increase between 2020 and 2021 highlights the impact of the COVID-19 pandemic, which has led to a surge in pneumothorax-related deaths and temporarily reversed the progress made previously with partial reversion by 2023. Notably, the mortality rates for spontaneous pneumothorax in 2020 (excluding COVID-related pneumothorax) were lower than pre-2020 mean; this was driven by a reduction in SSP deaths (Fig. 2). In 2020, the UK had 2 periods of COVID-19 lockdown and patients with underlying lung disease were strongly advised to “shield”.^11^ This led to a reduction in infections/exacerbations of underlying lung disease events.^12^^,^^13^ Therefore, it is likely that the reduction in mortality for non-COVID SP was driven by fewer exacerbations. In 2021–23, the mortality rates for non-COVID spontaneous pneumothorax returned to pre-2020 levels.

The association of pneumothorax with COVID-19 is well described in hospitalised patients.^14^ A systematic review of nine observational studies found an overall incidence of pneumothorax of 0.3%, which increased to 12.8–23.8% in patients requiring invasive ventilation, as COVID-19-related pneumothorax is likely to signify more severe disease.^15^ Mortality rates reported varied from 21.0 to 88.3%.^16^

In our in-hospital analyses, ventilatory support was substantially more common in COVID-related than non-COVID deaths (particularly within in-hospital PSP) supporting the interpretation that severe respiratory failure was a key driver of adverse outcomes in these patients (eTable S6). Because OPCS-4 procedure codes do not encode event order, we cannot determine whether ventilation preceded pneumothorax or vice versa; these results therefore quantify ventilation during the terminal admission and should not be taken as proof of causality. In a secondary analysis of all emergency admissions for spontaneous pneumothorax, we found that when PSP or SSP was listed as the main/primary diagnosis (indicating that the main reason for hospitalisation was for pneumothorax), the in-hospital mortality risk was relatively low. Even when considering all-cause in-hospital deaths among these admissions, patients whose primary reason for admission was spontaneous pneumothorax rarely died because of the pneumothorax itself; if death occurred, it was usually attributable to other serious conditions such as pneumonia, cancer, or cardiovascular disease. This analysis (illustrated in Fig. 3 and detailed Table 1) provides a more comprehensive picture of outcomes following a spontaneous pneumothorax admission. It shows that comparatively few emergency spontaneous pneumothorax admissions result in an in-hospital death, and deaths specifically caused by pneumothorax during those admissions are rare, but all-cause in-hospital mortality for those with a spontaneous pneumothorax-related admission is significant older patients, particularly in those with SSP.

The last study of mortality rates in England found a marked increase in the death rate for pneumothorax in patients aged 55 and over years between 1960 and 1990, with a subsequent steep decline in the 1990s.^3^ This was not easily explainable by a decline in diagnosis or deaths of underlying lung disease such as COPD or emphysema.^3^ Compared with the earlier report, this study examined death certificates and linked HES-APC records to split in-hospital spontaneous pneumothorax-related deaths into PSP and SSP, showing that annual mortality averaged approximately 1.0 per million for PSP and 4.3 per million for SSP. This suggests that while overall pneumothorax mortality has evolved since 1997, secondary pneumothorax (often associated with underlying lung disease) continues to account for a higher mortality rate than primary pneumothorax.

In both in-hospital PSP and SSP patients, the mortality rate was highest in the 65+ age group. This is consistent with previous literature on risk factors for inpatient mortality. A US study of PSP patients from 2016 to 2019 (looking at cannabis vs non-cannabis users) found the strongest predictors of inpatient mortality were need for invasive mechanical ventilation, sudden cardiac arrest and age 70+ years.^17^ A US study using the US Readmission dataset (2016–2017) found in-hospital, 30-day, 90-day, and 1-year mortality rates for SSP were 3.74%, 4.29%, 5.95%, and 9.13%, respectively^18^; patients who had a recurrence prevention procedure (medical/chemical or surgical pleurodesis) had a lower readmission rate and lower 30-day and 1 year mortality. An earlier analysis of the same US dataset (2012–2013) found overall inpatient mortality was 3.1% on the index admission with pneumothorax and 4.6% on readmissions.^19^ No PSP vs SSP breakdown was provided, but risk factors for mortality were older age and concurrent cancer diagnosis.^19^ Analysis of a German admissions dataset (2011–2015) found that inpatient mortality in the age group 15–40 years was 0.06–0.32%, compared to 8–18% in the age group 70–95 years.^20^ Several smaller single-centre studies from Japan confirmed higher inpatient mortality rates for SSP than PSP.^21^, ^22^, ^23^

Our findings underscore the importance of optimising management strategies to reduce the risk of pneumothorax and improve outcomes. Historically, advances in respiratory care have significantly lowered both incidence and mortality: before the pandemic, adopting lung-protective ventilation for Acute Respiratory Distress Syndrome (ARDS), using low tidal volumes and limited airway pressures, reduced ventilator-related pneumothorax rates from 55% to 17%.^24^ Similar attention to ventilatory management in COVID-19, including strict use of Acute Respiratory Distress Syndrome Network (ARDSNet) protocols, timely proning, and maintaining safe pressures, appears crucial for minimising barotrauma. Early consideration of extracorporeal membrane oxygenation in refractory COVID-19 ARDS may also help prevent air leak complications, although current evidence remains preliminary. In case series of COVID-19 related pneumothorax, the majority of patients (71%) were managed with chest drain^14^; however, the optimal management has not been defined.

Distinguishing primary from secondary spontaneous pneumothorax at the population level is intrinsically challenging. We therefore defined PSP as no recorded chronic lung disease in the death certificate or linked HES-APC record (73 ICD-10 codes; eTable S1), with all other cases classified as SSP. This approach aligns with our previous national work.^1^^,^^5^ Whilst the accuracy of coding for respiratory comorbidities is good in HES-APC^7^; smoking status is not. Therefore, our definition of PSP is not identical to clinical guideline definitions (e.g., younger age, limited smoking history, absence of significant lung disease). Two implications follow. First, out-of-hospital deaths without hospital records are more susceptible to misclassification if underlying lung disease was undocumented; accordingly, we emphasise in-hospital analyses. Second, if the denominator is changed from the general population to all emergency admissions for PSP, the estimated PSP mortality is low, reflecting the rarity of fatal PSP; this aligns with the age distribution observed in our previous hospitalisation study, in which PSP incidence peaked in younger age groups. Regarding extra-pulmonary comorbidity, cardiovascular disease consistently featured as the leading non-respiratory underlying-cause category for in-hospital spontaneous pneumothorax deaths across periods, particularly among older adults and in SSP. This supports the view that pneumothorax often co-occurs with substantial systemic disease burden and that the pathway to death may be dominated by comorbid conditions even when pneumothorax lies within the causal chain of admission.

A strength of our study is the use of multiple-cause mortality records, which capture any mention of pneumothorax on the death certificate rather than relying exclusively on a single underlying cause of death. This approach provides a much more comprehensive measure of spontaneous pneumothorax-related mortality. Most traditional mortality analyses consider only underlying cause, which would severely underestimate the total contribution of spontaneous pneumothorax to the premature mortality burden.^25^ In our data, only about 8% of spontaneous pneumothorax-related deaths listed pneumothorax as the underlying cause. However, pneumothorax appeared in Part I of the death certificate, the section listing the direct chain of events leading to death, in 64% of cases; in 36% it was recorded in Part II as a significant contributing condition (eTable S2). From a practical perspective, if we had focused only on underlying-cause data, we would have missed the vast majority of cases where pneumothorax contributed to mortality. This issue is especially pronounced during the COVID-19 pandemic period: many patients who died with COVID-19 experienced a pneumothorax as a complication, but their death certificates often listed COVID-19 as the underlying cause. Without a multiple-cause analysis, these pneumothorax-related deaths would not be counted at all in pneumothorax mortality statistics, thereby masking the pandemic's true impact on this condition.

This study has several limitations. First, in-hospital PSP and SSP were distinguished solely by linking death certificates to prior HES-APC records to identify chronic lung disease. For out-of-hospital deaths, especially those with little or no other historic hospital admission data, disease history could not reliably be captured because primary care records were not linked. However, since most spontaneous pneumothorax-related deaths occurred in hospital, we have maximised the accuracy of capturing the relative PSP and SSP burden by restricting these definitions to in-hospital deaths. Distinguishing Part I from Part II of the death certificate was only possible from 2017; therefore, most analyses were based on whether pneumothorax was recorded as the underlying cause of death or mentioned anywhere on the death certificate (Part I or II). Despite this constraint, the clear difference between deaths in which pneumothorax was the underlying cause and those in which it was noted elsewhere supports the value of analysing multiple causes of death rather than the underlying cause alone. Changes in clinician awareness and certification practices over time may affect the recording of pneumothorax; however, concordance between the leading primary diagnosis at the terminal admission and the underlying cause of death recorded on the death certificate can support the completeness and consistency of both datasets. Although procedure (OPCS-4) codes in HES-APC are precisely dated, the diagnosis codes (ICD-10) lack temporal resolution beyond the hospital episode start and end dates. This precludes ascertaining, for example, the order of ventilation and pneumothorax. Procedure codes may also under-record ward-level NIV. Our estimates therefore reflect ventilation during the terminal admission rather than causal sequencing. In addition, specific infective aetiologies that predispose to pneumothorax (e.g., influenza, Pneumocystis) are infrequently and inconsistently captured in national registrations and hospital coding, with small cell counts, so we did not model them as separate strata. However, we do fully present data on pneumonia as a co-diagnosis.

### Conclusion

Prior to 2020, the mortality rate for spontaneous pneumothorax had been steadily declining, which is consistent with improvements in respiratory care and service delivery over time, alongside demographic changes and the impact of COVID-19. However, the outbreak of the COVID-19 pandemic led to a notable increase in spontaneous pneumothorax-related mortality rates, highlighting how external factors can disrupt positive trends. Relying solely on underlying cause of death substantially underestimates pneumothorax's overall contribution to mortality in England and supports the routine use of multiple-cause mortality analyses for conditions such as spontaneous pneumothorax. Elderly patients, especially those over 65 years of age with chronic lung disease (such as COPD), had the highest risk of spontaneous pneumothorax-related mortality, underscoring the necessity of closely managing this vulnerable population. It is worth noting that spontaneous pneumothorax occurring in patients without known lung disease is not entirely benign, as it can still lead to death in patients, particularly in those over the age of 65 years; although the absolute risk remains low in younger age groups. These findings underscore the importance of recognising pneumothorax, which can occur in conjunction with other chronic disease, as a marker of potential poor outcomes and enhancing treatment strategies to continue improving patient outcomes.

## Contributors

All authors included on the paper fulfil the criteria of authorship. RH proposed the study. XZ and RG designed the methodology and statistical strategy. RH conducted the literature search. XZ conducted the data analysis and generated the figures. All authors interpreted the data. XZ and RH draughted the manuscript with contributions from RG and EM. XZ and RG had full access to all the data in the study and take responsibility for the integrity of the data and the accuracy of the data analysis. RG verified the data. All authors had final responsibility for decision to submit for publication.

## Data sharing statement

All data used in this study are available through application to NHS England at https://digital.nhs.uk/.

## Declaration of interests

None to declare.
