# Supplementary material for: Trends in pneumothorax mortality in England (2004–2023): a population-based observational study
Source: Lancet Reg Health Eur. 2026 Mar 4;64:101632. doi: 10.1016/j.lanepe.2026.101632 (PMC12972727; doi:10.1016/j.lanepe.2026.101632)
Supplement: Supplementary Figures and Tables [file mmc1.pdf]

Supplementary material

eTable 1 ICD-10 codes for any diagnosis of chronic lung disease 2
eTable 2 Spontaneous Pneumothorax Mortality in England between 2017 and 2023, age 15 Years and Older, by cause of death, position in death certificate.. 3
eTable 3 Leading primary diagnoses (ICD-10 three-character codes) among in-hospital SP-related deaths in England, by period 2004–07, 2016–19, and 2020–23. Sections: A) SP (all), B) PSP, C) SSP; all ages. 4
eTable 4 Leading underlying causes (ICD-10 three-character codes) of in-hospital SP-related deaths among people aged ⩾50 years in England, by period 2004–07, 2016–19, and 2020–23. Sections: (A) SP (all), (B) PSP, (C) SSP. 5
eTable 5 Leading primary diagnoses (ICD-10 three-character codes) among in-hospital SP-related deaths in England among people aged ⩾50 years, by period 2004–07, 2016–19, and 2020–23. Sections: (A) SP (all), (B) PSP, (C) SSP 6
eTable 6 Breathing support and non-invasive ventilation (NIV) among in-hospital SP-related deaths by PSP vs SSP and COVID-19 involvement, England (Jan 2004–Dec 2023) 7

eFigure 1 Flow diagram of deaths included in the analysis with Spontaneous Pneumothorax from 2003 to 2023 9
eFigure 2 Adjusted incidence rate ratios (IRRs) for SP-related mortality by period in England (pre-2019 vs 2020–2023): SP (all), in-hospital SP, in-hospital PSP, and in-hospital SSP; with and without COVID-19. 10
eFigure 3 Age-standardised mortality rates for SP-related deaths by sex and age group in England (Jan 2004–Dec 2023). Panels: (a) SP (all deaths); (b) In-hospital SP; (c) In-hospital PSP (non-SSP); (d) In-hospital SSP. 13

eTable 1 ICD-10 codes for any diagnosis of chronic lung disease

Tuberculosis	A150, A151, A152, A153, A155, A156, A157, A158, A159, A160, A161, A162, A164, A165, A168, A169, B909
Malignancy	C340, C341, C342, C343, C348, C349, C780, D020, D022, D023, D143, D381, D382, Z851, Z852
Sarcoidosis	D860, D861, D862, D863, D868, D869
Cystic Fibrosis	E840, E841, E848, E849
COPD/Emphysema	J40X, J410, J411, J42X, J430, J431, J432, J438, J439, J440, J441, J448, J449
Asthma	J450, J451, J458, J459, J47X
Interstitial lung disease	J700, J701, J703, J704, J708, J709, J840, J841, J848, J849, J961, J991, J998

eTable 2 Spontaneous Pneumothorax Mortality in England between 2017 and 2023, age 15 Years and Older, by cause of death, position in death certificate.

Position in death certificate	Number	Percentage of total
Underlying Cause of Death	215	7.9%
Part I	1750	64.0%
Part II	985	36.0%
Underlying Cause of Death or Part I	1790	65.6%
Any mention	2735	100%

Part I lists the causal sequence of conditions leading directly to death; Part II lists other significant conditions contributing to death but not part of the direct causal sequence

eTable 3 Leading primary diagnoses (ICD-10 three-character codes) among in-hospital SP-related deaths in England, by period 2004–07, 2016–19, and 2020–23. Sections: A) SP (all), B) PSP, C) SSP; all ages.

**A. All age, in-hospital SP-related death**											
**Leading primary diagnosis in 2004-07**				**Leading primary diagnosis in 2016-19**				**Leading primary diagnosis in 2020-23**			
ICD-10	Description	N	%	ICD-10	Description	N	%	ICD-10	Description	N	%
J93	Pneumothorax	341	34.3	J93	Pneumothorax	415	42.3	J93	Pneumothorax	397	36.6
J18	Pneumonia, organism unspecified	98	9.9	J18	Pneumonia, organism unspecified	164	16.7	U07	COVID-19	155	14.3
J44	Other chronic obstructive pulmonary disease	94	9.5	J44	Other chronic obstructive pulmonary disease	74	7.6	J18	Pneumonia, organism unspecified	118	10.9
C34	Malignant neoplasm of bronchus and lung	33	3.3	A41	Other sepsis	30	3.1	J44	Other chronic obstructive pulmonary disease	59	5.4
J96	Respiratory failure, not elsewhere classified	25	2.5	J96	Respiratory failure, not elsewhere classified	22	2.2	J96	Respiratory failure, not elsewhere classified	31	2.9
R69	Unknown and unspecified causes of morbidity	24	2.4	J84	Other interstitial pulmonary diseases	20	2	A41	Other sepsis	27	2.5
J84	Other interstitial pulmonary diseases	18	1.8	J94	pleural conditions	14	1.4	J94	pleural conditions	21	1.9
J43	Emphysema	17	1.7	J69	Pneumonitis due to food and vomit	9	0.9	J84	Other interstitial pulmonary diseases	20	1.8
J22	Unspecified acute lower respiratory infection	14	1.4	I46	Cardiac arrest	8	0.8	C34	Malignant neoplasm of bronchus and lung	15	1.4
I50	Heart failure	13	1.3	J43	Emphysema	8	0.8	I46	Cardiac arrest	10	0.9
**B. All age, in-hospital PSP-related death**											
**Leading primary diagnosis in 2004-07**				**Leading primary diagnosis in 2016-19**				**Leading primary diagnosis in 2020-23**			
ICD-10	Description	N	%	ICD-10	Description	N	%	ICD-10	Description	N	%
J93	Pneumothorax	58	24.4	J18	Pneumonia, organism unspecified	37	22.2	U07	COVID-19	64	32.8
J18	Pneumonia, organism unspecified	31	13	J93	Pneumothorax	34	20.4	J93	Pneumothorax	27	13.8
R69	Unknown and unspecified causes of morbidity	9	3.8	A41	Other sepsis	10	6	J18	Pneumonia, organism unspecified	21	10.8
**C. All age, in-hospital SSP-related death**											
**Leading primary diagnosis in 2004-07**				**Leading primary diagnosis in 2016-19**				**Leading primary diagnosis in 2020-23**			
ICD-10	Description	N	%	ICD-10	Description	N	%	ICD-10	Description	N	%
J93	Pneumothorax	283	37.5	J93	Pneumothorax	381	46.9	J93	Pneumothorax	370	41.6
J44	Other chronic obstructive pulmonary disease	94	12.5	J18	Pneumonia, organism unspecified	127	15.6	J18	Pneumonia, organism unspecified	97	10.9
J18	Pneumonia, organism unspecified	67	8.9	J44	Other chronic obstructive pulmonary disease	74	9.1	U07	COVID-19	91	10.2
C34	Malignant neoplasm of bronchus and lung	33	4.4	A41	Other sepsis	20	2.5	J44	Other chronic obstructive pulmonary disease	59	6.6
J96	Respiratory failure, not elsewhere classified	24	3.2	J84	Other interstitial pulmonary diseases	20	2.5	J96	Respiratory failure, not elsewhere classified	27	3
J84	Other interstitial pulmonary diseases	18	2.4	J96	Respiratory failure, not elsewhere classified	18	2.2	J84	Other interstitial pulmonary diseases	20	2.2
J43	Emphysema	17	2.3	J94	pleural conditions	10	1.2	A41	Other sepsis	19	2.1
R69	Unknown and unspecified causes of morbidity	15	2	J43	Emphysema	8	1	C34	Malignant neoplasm of bronchus and lung	15	1.7
J22	Unspecified acute lower respiratory infection	12	1.6	I46	Cardiac arrest	7	0.9	J94	pleural conditions	14	1.6
I46	Cardiac arrest	10	1.3	C34	Malignant neoplasm of bronchus and lung	6	0.7	J43	Emphysema	10	1.1

Primary diagnosis refers to the hospital primary diagnosis recorded in HES APC (ICD-10, three-character level). N = number of in-hospital SP-related deaths in the period; % = percentage of all such deaths within that period. PSP = primary spontaneous pneumothorax; SSP = secondary spontaneous pneumothorax. COVID-19 is ICD-10 U07. Periods cover January 2004 to December 2023.

eTable 4 Leading underlying causes (ICD-10 three-character codes) of in-hospital SP-related deaths among people aged ≥50 years in England, by period 2004–07, 2016–19, and 2020–23. Sections: (A) SP (all), (B) PSP, (C) SSP.

**A. Age 50+, in-hospital SP-related death**											
**Leading underlying cause in 2004-07**				**Leading underlying cause in 2016-19**				**Leading underlying cause in 2020-23**			
ICD-10	Description	N	%	ICD-10	Description	N	%	ICD-10	Description	N	%
J44	Other chronic obstructive pulmonary disease	321	34.3	J44	Other chronic obstructive pulmonary disease	343	36.3	J44	Other chronic obstructive pulmonary disease	308	29.6
J93	Pneumothorax	119	12.7	J84	Other interstitial pulmonary diseases	96	10.2	U07	COVID-19	202	19.4
C34	Malignant neoplasm of bronchus and lung	71	7.6	J43	Emphysema	88	9.3	J84	Other interstitial pulmonary diseases	92	8.9
J84	Other interstitial pulmonary diseases	67	7.2	J93	Pneumothorax	63	6.7	J43	Emphysema	90	8.7
J43	Emphysema	56	6.0	C34	Malignant neoplasm of bronchus and lung	52	5.5	J93	Pneumothorax	74	7.1
I25	Chronic ischaemic heart disease	19	2.0	J18	Pneumonia, organism unspecified	42	4.4	C34	Malignant neoplasm of bronchus and lung	39	3.8
C15	Malignant neoplasm of oesophagus	13	1.4	F03	Unspecified dementia	13	1.4	J47	Bronchiectasis	11	1.1
I21	Acute myocardial infarction	12	1.3	I21	Acute myocardial infarction	12	1.3	C15	Malignant neoplasm of oesophagus	8	0.8
I64	Stroke, not specified as haemorrhage or infarction	12	1.3	I64	Stroke, not specified as haemorrhage or infarction	12	1.3	I21	Acute myocardial infarction	7	0.7
J47	Bronchiectasis	12	1.3	J86	Pyothorax	8	0.8	I25	Chronic ischaemic heart disease	7	0.7
**B. Age 50+, in-hospital PSP-related death**											
Leading underlying cause in 2004-07				**Leading underlying cause in 2016-19**				**Leading underlying cause in 2020-23**			
ICD-10	Description	N	%	ICD-10	Description	N	%	ICD-10	Description	N	%
J93	Pneumothorax	82	37.6	J18	Pneumonia, organism unspecified	29	18.8	U07	COVID-19	69	38.3
I25	Chronic ischaemic heart disease	12	5.5	J93	Pneumothorax	29	18.8	J93	Pneumothorax	44	24.4
				F03	Unspecified dementia	10	6.5				
**C. Age 50+, in-hospital SSP-related death**											
**Leading primary diagnosis in 2004-07**				**Leading primary diagnosis in 2016-19**				**Leading underlying cause in 2004-07**			
ICD-10	Description	N	%	ICD-10	Description	N	%	ICD-10	Description	N	%
J44	Other chronic obstructive pulmonary disease	321	44.7	J44	Other chronic obstructive pulmonary disease	343	43.4	J44	Other chronic obstructive pulmonary disease	308	35.9
C34	Malignant neoplasm of bronchus and lung	71	9.9	J84	Other interstitial pulmonary diseases	96	12.2	U07	COVID-19	133	15.5
J84	Other interstitial pulmonary diseases	67	9.3	J43	Emphysema	88	11.1	J84	Other interstitial pulmonary diseases	92	10.7
J43	Emphysema	56	7.8	C34	Malignant neoplasm of bronchus and lung	52	6.6	J43	Emphysema	90	10.5
J93	Pneumothorax	37	5.2	J93	Pneumothorax	34	4.3	C34	Malignant neoplasm of bronchus and lung	39	4.5
J47	Bronchiectasis	12	1.7	J18	Pneumonia, organism unspecified	13	1.6	J93	Pneumothorax	30	3.5
I64	Stroke, not specified as haemorrhage or infarction	10	1.4	I21	Acute myocardial infarction	8	1.0	J47	Bronchiectasis	11	1.3
I21	Acute myocardial infarction	9	1.3	C50	Malignant neoplasm of breast	7	0.9	I21	Acute myocardial infarction	6	0.7
J98	Other respiratory disorders	9	1.3	I64	Stroke, not specified as haemorrhage or infarction	7	0.9	J45	Asthma	6	0.7
C15	Malignant neoplasm of oesophagus	8	1.1	I25	Chronic ischaemic heart disease	6	0.8	J67	Hypersensitivity pneumonitis due to organic dust	6	0.7

Footnote. Underlying cause is taken from the death registration (ICD-10, three-character level). N = number of in-hospital SP-related deaths in the subgroup and period; % = percentage of all such deaths within that subgroup and period. PSP = primary spontaneous pneumothorax; SSP = secondary spontaneous pneumothorax; COVID-19 is ICD-10 U07. Periods cover January 2004 to December 2023.

eTable 5 Leading primary diagnoses (ICD-10 three-character codes) among in-hospital SP-related deaths in England among people aged ≥50 years, by period 2004–07, 2016–19, and 2020–23. Sections: (A) SP (all), (B) PSP, (C) SSP.

**A. Age 50+, in-hospital SP-related death**											
**Leading primary diagnosis in 2004-07**				**Leading primary diagnosis in 2016-19**				**Leading primary diagnosis in 2020-23**			
ICD-10	Description	N	%	ICD-10	Description	N	%	ICD-10	Description	N	%
J93	Pneumothorax	327	34.9	J93	Pneumothorax	409	43.3	J93	Pneumothorax	387	37.2
J44	Other chronic obstructive pulmonary disease	93	9.9	J18	Pneumonia, organism unspecified	156	16.5	U07	COVID-19	147	14.1
J18	Pneumonia, organism unspecified	91	9.7	J44	Other chronic obstructive pulmonary disease	73	7.7	J18	Pneumonia, organism unspecified	117	11.3
C34	Malignant neoplasm of bronchus and lung	33	3.5	A41	Other sepsis	29	3.1	J44	Other chronic obstructive pulmonary disease	59	5.7
J96	Respiratory failure, not elsewhere classified	22	2.4	J96	Respiratory failure, not elsewhere classified	20	2.1	J96	Respiratory failure, not elsewhere classified	30	2.9
R69	Unknown and unspecified causes of morbidity	21	2.2	J84	Other interstitial pulmonary diseases	19	2.0	A41	Other sepsis	24	2.3
J84	Other interstitial pulmonary diseases	18	1.9	J94	pleural conditions	14	1.5	J94	pleural conditions	21	2.0
J43	Emphysema	17	1.8	J43	Emphysema	8	0.8	J84	Other interstitial pulmonary diseases	17	1.6
J22	Unspecified acute lower respiratory infection	14	1.5	S72	Fracture of femur	8	0.8	C34	Malignant neoplasm of bronchus and lung	14	1.3
I50	Heart failure	13	1.4	I46	Cardiac arrest	7	0.7	I46	Cardiac arrest	10	1.0
**B. Age 50+, in-hospital PSP-related death**											
**Leading primary diagnosis in 2004-07**				**Leading primary diagnosis in 2016-19**				**Leading primary diagnosis in 2020-23**			
ICD-10	Description	N	%	ICD-10	Description	N	%	ICD-10	Description	N	%
J93	Pneumothorax	53	24.3	J18	Pneumonia, organism unspecified	36	23.4	U07	COVID-19	60	33.3
J18	Pneumonia, organism unspecified	28	12.8	J93	Pneumothorax	33	21.4	J93	Pneumothorax	25	13.9
R69	Unknown and unspecified causes of morbidity	8	3.7	A41	Other sepsis	9	5.8	J18	Pneumonia, organism unspecified	21	11.7
**C. Age 50+, in-hospital SSP-related death**											
**Leading primary diagnosis in 2004-07**				**Leading primary diagnosis in 2016-19**				**Leading primary diagnosis in 2020-23**			
ICD-10	Description	N	%	ICD-10	Description	N	%	ICD-10	Description	N	%
J93	Pneumothorax	274	38.2	J93	Pneumothorax	376	47.6	J93	Pneumothorax	362	42.1
J44	Other chronic obstructive pulmonary disease	93	13.0	J18	Pneumonia, organism unspecified	120	15.2	J18	Pneumonia, organism unspecified	96	11.2
J18	Pneumonia, organism unspecified	63	8.8	J44	Other chronic obstructive pulmonary disease	73	9.2	U07	COVID-19	87	10.1
C34	Malignant neoplasm of bronchus and lung	33	4.6	A41	Other sepsis	20	2.5	J44	Other chronic obstructive pulmonary disease	59	6.9
J96	Respiratory failure, not elsewhere classified	21	2.9	J84	Other interstitial pulmonary diseases	19	2.4	J96	Respiratory failure, not elsewhere classified	26	3.0
J84	Other interstitial pulmonary diseases	18	2.5	J96	Respiratory failure, not elsewhere classified	17	2.2	A41	Other sepsis	18	2.1
J43	Emphysema	17	2.4	J94	pleural conditions	10	1.3	J84	Other interstitial pulmonary diseases	17	2.0
R69	Unknown and unspecified causes of morbidity	13	1.8	J43	Emphysema	8	1.0	C34	Malignant neoplasm of bronchus and lung	14	1.6
J22	Unspecified acute lower respiratory infection	12	1.7	I46	Cardiac arrest	7	0.9	J94	pleural conditions	14	1.6
R06	Abnormalities of breathing	10	1.4	C34	Malignant neoplasm of bronchus and lung	6	0.8	J43	Emphysema	10	1.2

Footnote. Primary diagnosis refers to the hospital primary diagnosis recorded in HES APC (ICD-10, three-character level). N = number of in-hospital SP-related deaths in the subgroup and period; % = percentage of all such deaths within that subgroup and period. PSP = primary spontaneous pneumothorax; SSP = secondary spontaneous pneumothorax; COVID-19 is ICD-10 U07. Periods cover January 2004 to December 2023.

eTable 6 Breathing support and non-invasive ventilation (NIV) among in-hospital SP-related deaths by PSP vs SSP and COVID-19 involvement, England (Jan 2004–Dec 2023)

Cohort	COVID status	Deaths (n)	Breathing support (n)	Breathing support (%)	NIV (n)	NIV (%)
**PSP**	Non-COVID	880	120	13.6	62	7.0
**PSP**	COVID-related	82	46	56.1	10	12.2
**SSP**	Non-COVID	3927	726	18.5	559	14.2
**SSP**	COVID-related	166	64	38.6	17	10.2

Footnote. Cohort indicates PSP (primary spontaneous pneumothorax) or SSP (secondary spontaneous pneumothorax). COVID status classifies deaths as COVID-related (ICD-10 U07 present on the death certificate) or non-COVID. Deaths (n) is the total number of in-hospital SP-related deaths in each cohort × COVID status subgroup (England, Jan 2004–Dec 2023). Breathing support (n, %) is flagged when any OPCS-4 procedure code E85* was recorded during the final hospital admission; NIV (n, %) when any OPCS-4 code E85.2 was recorded. Percentages are within-subgroup proportions. Missing procedure codes were treated as not present.

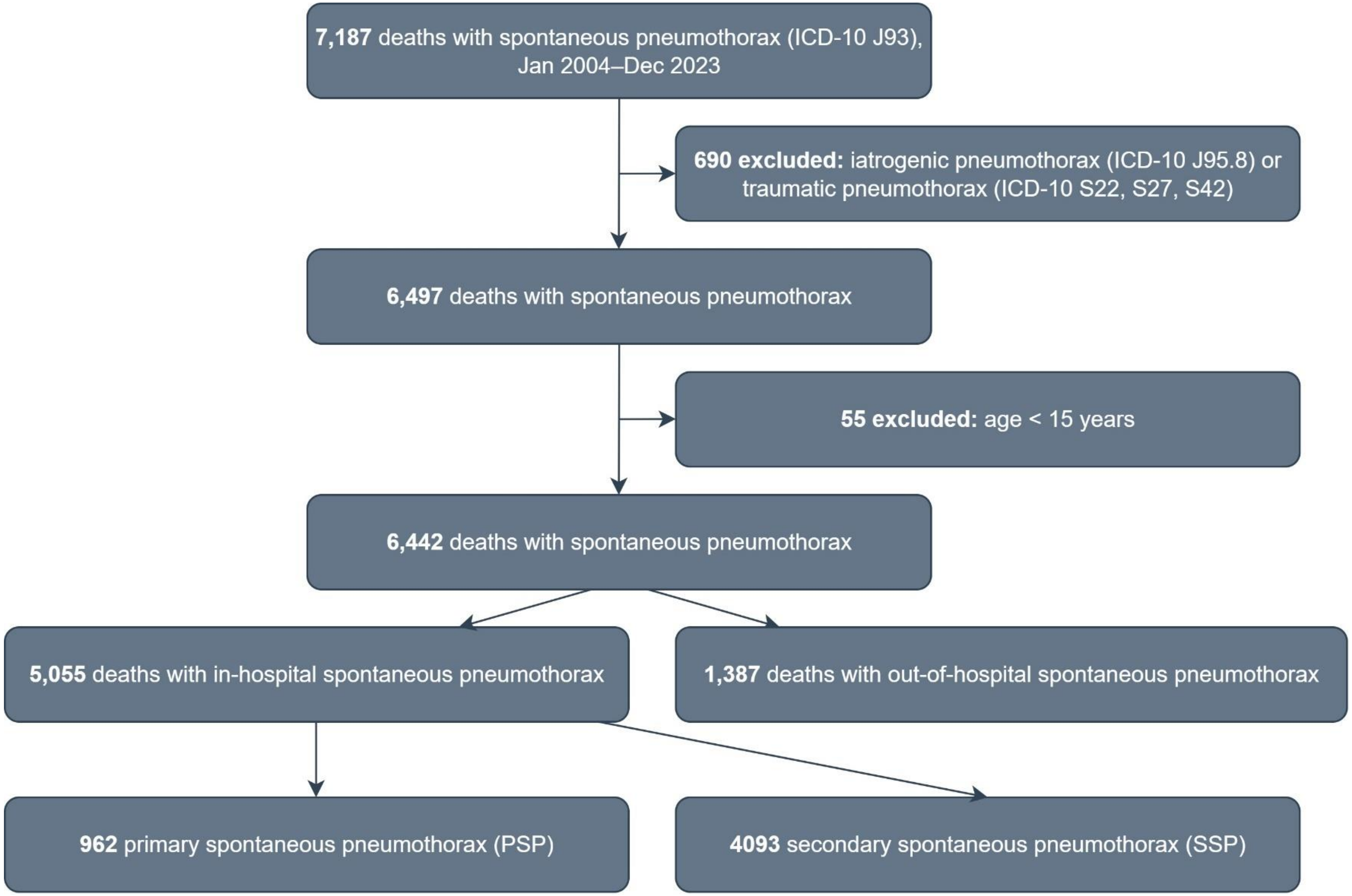
7,187 deaths with spontaneous pneumothorax (ICD-10 J93), Jan 2004–Dec 2023
690 excluded: iatrogenic pneumothorax (ICD-10 J95.8) or traumatic pneumothorax (ICD-10 S22, S27, S42)
6,497 deaths with spontaneous pneumothorax
55 excluded: age < 15 years
6,442 deaths with spontaneous pneumothorax
5,055 deaths with in-hospital spontaneous pneumothorax
1,387 deaths with out-of-hospital spontaneous pneumothorax
962 primary spontaneous pneumothorax (PSP)
4093 secondary spontaneous pneumothorax (SSP)

eFigure 1 Flow diagram of case selection for deaths with spontaneous pneumothorax (ICD-10 J93) in England, Jan 2004–Dec 2023

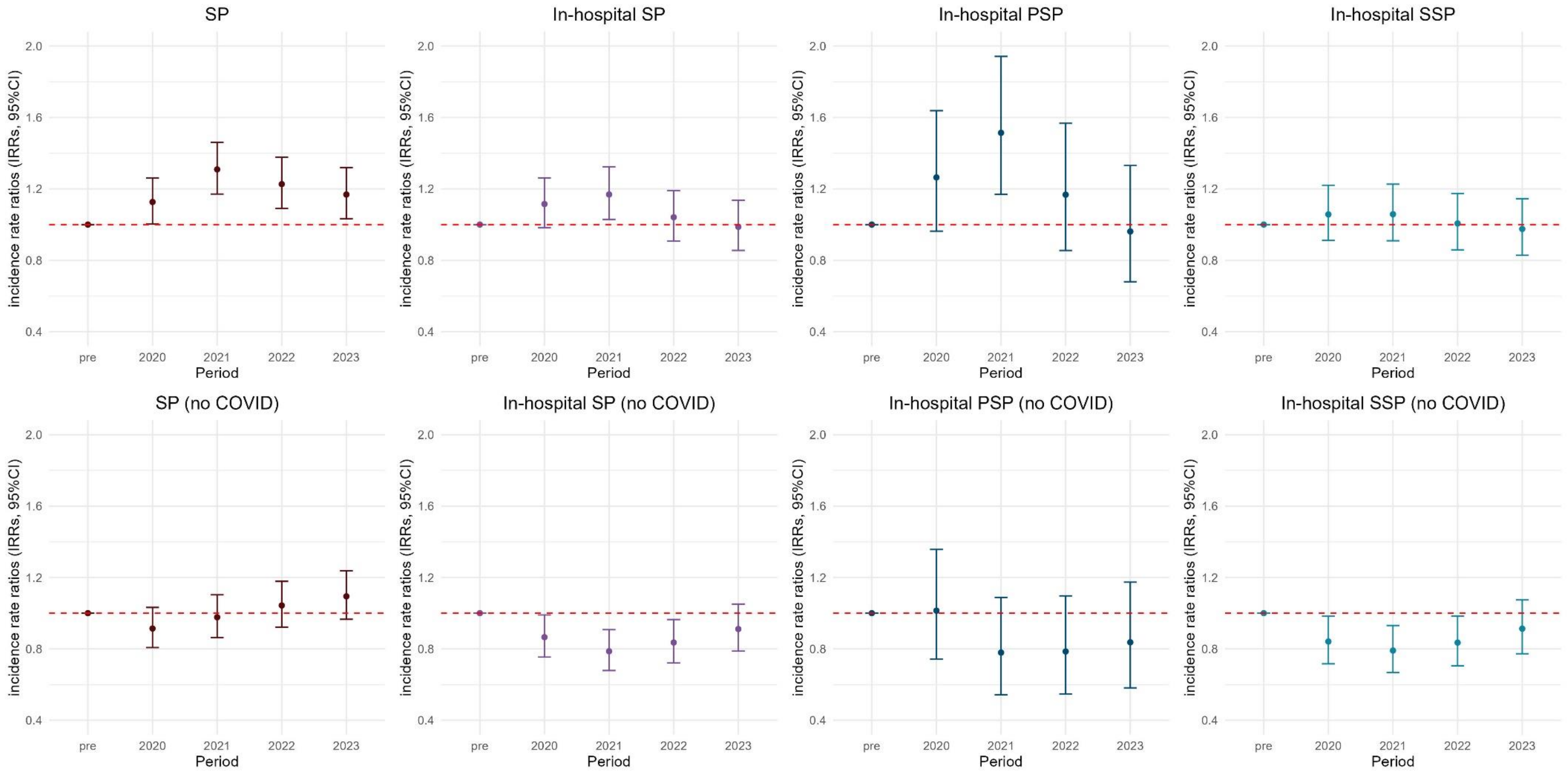

Footnote: Points show IRRs from quasi-Poisson models of annual age- and sex-specific mortality rates per 1,000,000, adjusting for age group, sex, and a linear time trend. The pre period (≤2019) is the reference (horizontal dashed line at IRR = 1). Error bars are 95% confidence intervals from the model. Top row includes all deaths; bottom row excludes deaths involving COVID-19 as defined in the Methods.

eFigure 2 Adjusted incidence rate ratios (IRRs) for SP-related mortality by period in England (pre-2019 vs 2020–2023): SP (all), in-hospital SP, in-hospital PSP, and in-hospital SSP; with and without COVID-19.

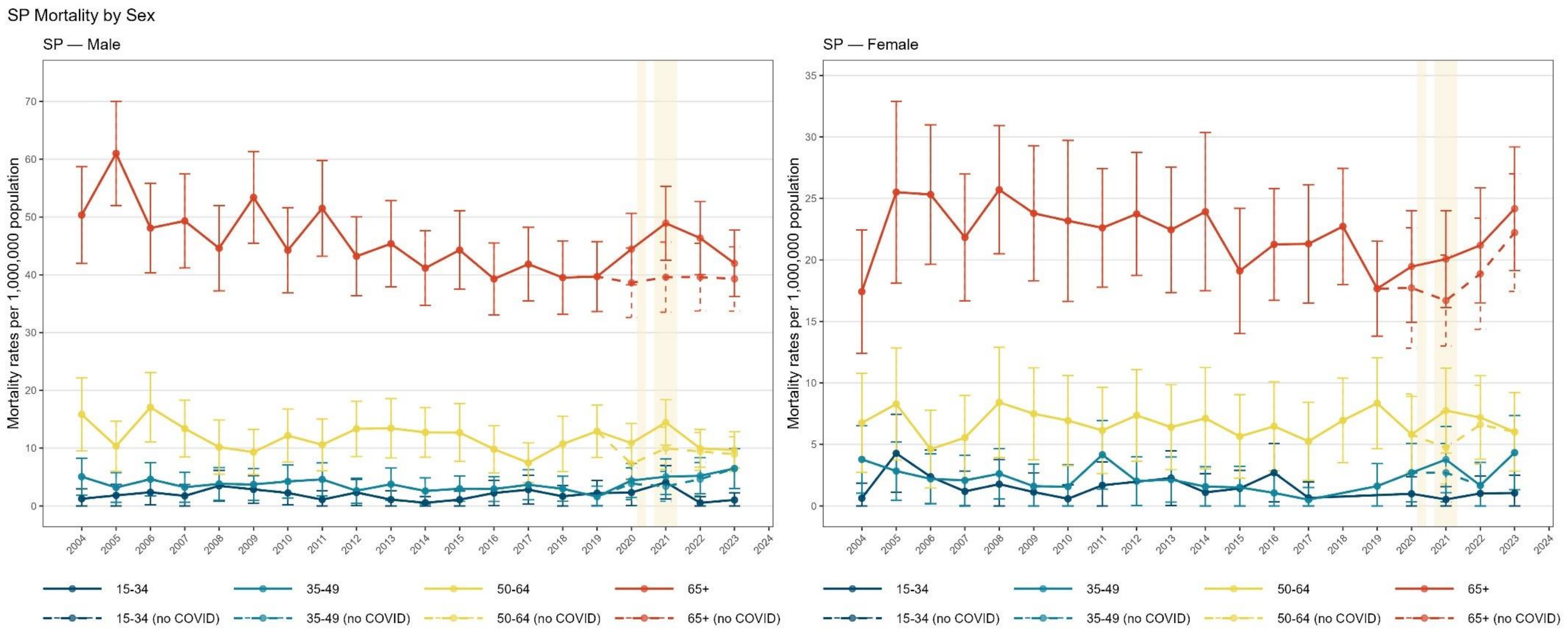

a.

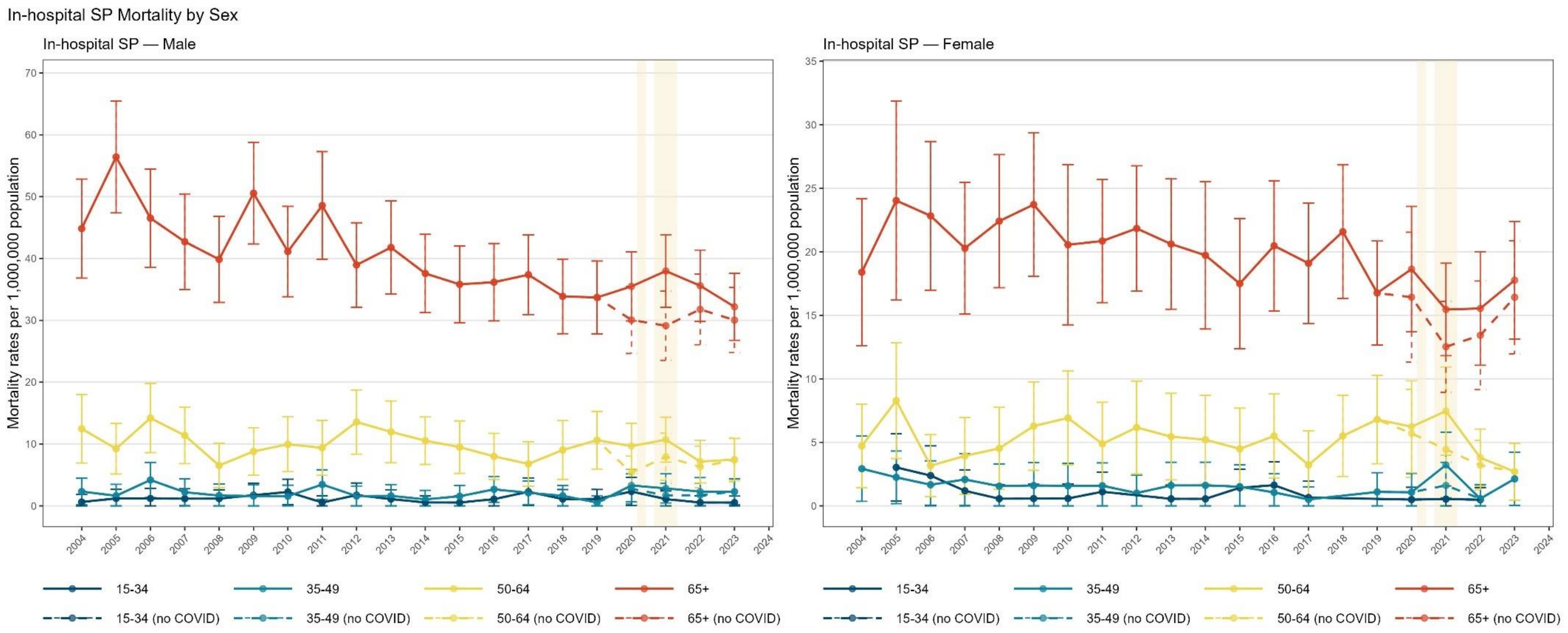

In-hospital SP Mortality by Sex
In-hospital SP — Male
In-hospital SP — Female
Mortality rates per 1,000,000 population
15-34
35-49
50-64
65+
15-34 (no COVID)
35-49 (no COVID)
50-64 (no COVID)
65+ (no COVID)

b.

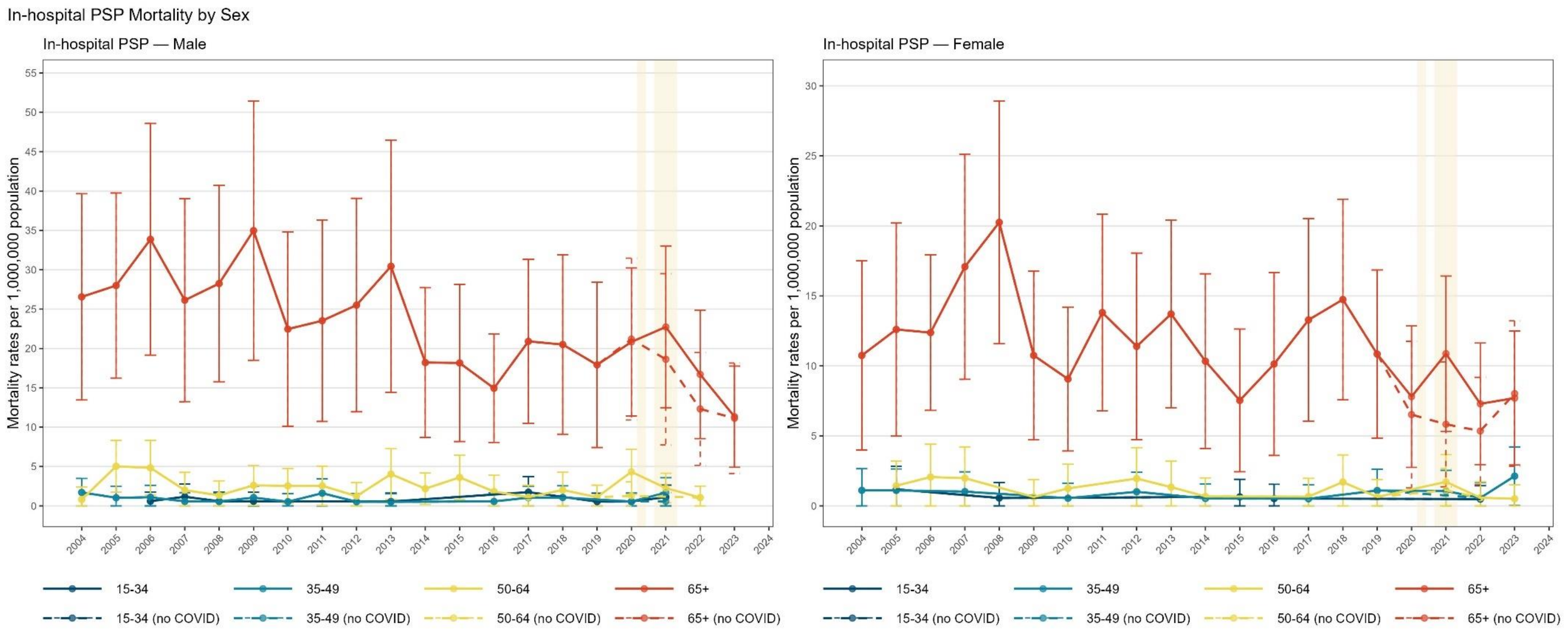
In-hospital PSP Mortality by Sex
In-hospital PSP — Male
In-hospital PSP — Female
Mortality rates per 1,000,000 population
15-34
35-49
50-64
65+
15-34 (no COVID)
35-49 (no COVID)
50-64 (no COVID)
65+ (no COVID)

c.

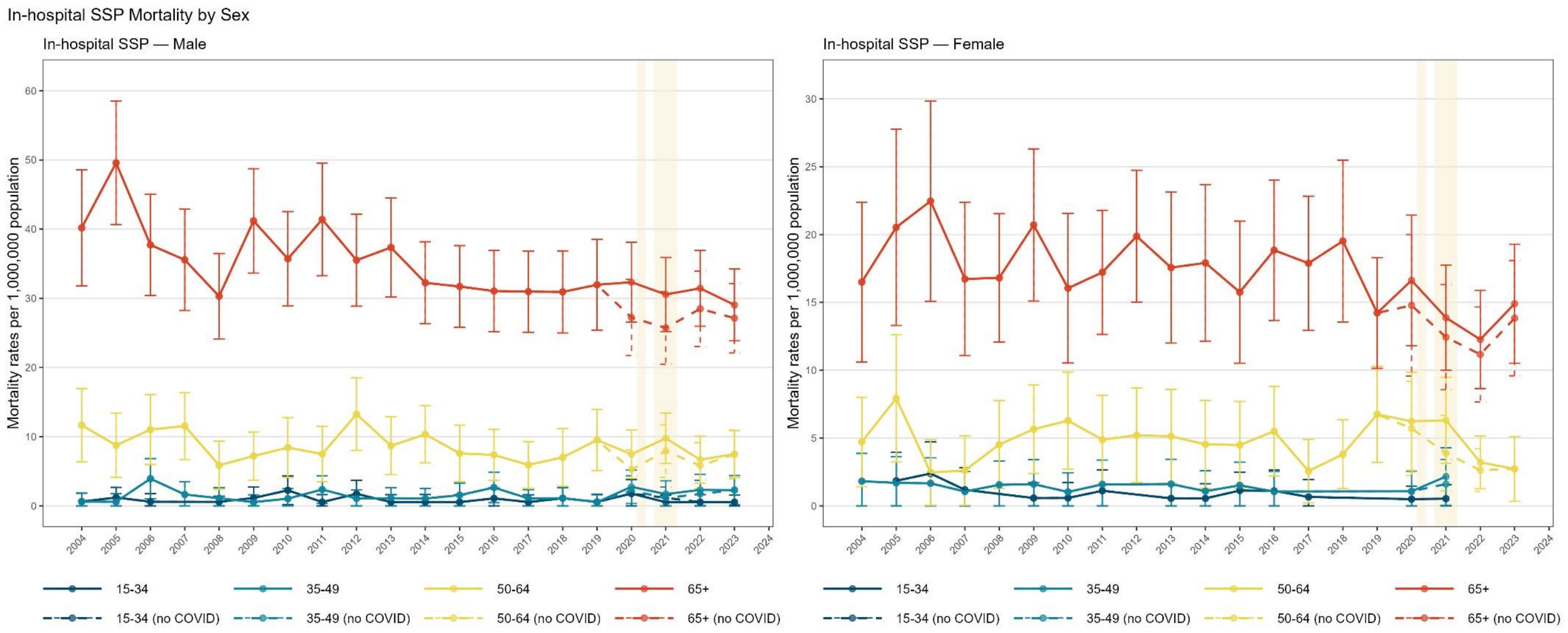

d.

Footnote: Points are directly age-standardised mortality rates per 1,000,000 population (standardised to the 2013 European Standard Population). Population denominators are ONS mid-year estimates of the usual resident population of England. Error bars show 95% confidence intervals from a Poisson approximation to the variance of the directly standardised rate and are truncated at zero where necessary. Colours indicate age groups (15–34, 35–49, 50–64, 65+); dashed lines ("no COVID") exclude deaths involving COVID-19 as defined in the Methods. Shaded bands indicate national lockdown periods in England. Each panel displays male (left) and female (right).

eFigure 3 Age-standardised mortality rates for SP-related deaths by sex and age group in England (Jan 2004–Dec 2023). Panels: (a) SP (all deaths); (b) In-hospital SP; (c) In-hospital PSP (non-SSP); (d) In-hospital SSP.
